# Epidemiology of carbapenem non-susceptible *Pseudomonas aeruginosa* isolates in Eastern Algeria

**DOI:** 10.1186/s13756-015-0067-2

**Published:** 2015-06-12

**Authors:** Samah Meradji, Abouddihaj Barguigua, Khalid Zerouali, Dekhil Mazouz, Houria Chettibi, Naima Elmdaghri, Mohammed Timinouni

**Affiliations:** Department of Biology, Biochemstry and Applied Microbiology Laboratory, Badji Mokhtar Faculty of sciences, Annaba University, Box 12, Sidi Amar, 23000 Annaba, Algeria; Molecular Bacteriology Laboratory, Pasteur Institute of Morocco, 1, Place Louis Pasteur, 20360 Casablanca,, Morocco; Microbiology Laboratory, Faculty of Medicine and Pharmacy, 1 Street Hospital, 20360 Casablanca, Morocco; Microbiology Laboratory, University Hospital Ibn Rochd, 23000 Annaba, Algeria

**Keywords:** *Pseudomonas aeruginosa*, Prevalence, Risk factors, Carbapenem resistance

## Abstract

**Background:**

Carbapenem resistance among *Pseudomonas aeruginosa* has become a serious life-threatening problem due to the limited therapeutic options. In this study, we investigated the prevalence and the molecular epidemiology of carbapenem resistant *Pseudomonas aeruginosa* (CRPA) isolated from three hospitals in Annaba city, Algeria.

**Methods:**

During the study period (January, 2012 to December, 2013), all patients infected by *P. aeruginosa* were considered as the potential study population. Antibiotic susceptibility testing was performed as recommended by the CLSI. Screening of carbapenemase producer isolates was performed by using imipenem-EDTA double-disk synergy test and modified Hodge test. CRPA isolates were tested for the presence of genes encoding β-lactamases, plasmid mediated quinolone resistance, aminoglycoside resistance and class 1 integrons were investigated by PCR and sequencing. The clonal relatedness among CRPA isolates was analyzed by pulsed-field gel electrophoresis method. The clinical data were collected to identify risk factors for CRPA carriage of *P. aeruginosa* infection.

**Results:**

The overall prevalence of CRPA was 18.75 %. The risk factors for carrying CRPA were the length of hospital stay (*p* = 0.04), co-infections with *Staphylococcus aureus* (*p* = 0.01), and the use of urinary catheter (*p =* 0.03). The in-hospital mortality rate among case patients was 13.33 % compared with 1.53 % for control patients (*p* = 0.09). All CRPA isolates were multidrug resistance and the most effective antibiotic against CRPA isolates was amikacin and colistin. PFGE revealed an epidemic clonal dissemination of CRPA isolates. None of CRPA isolated were found to be carbapenemase-producers. The *bla*_PSE-1_ and *aac(3)-II* gene was detected in two and five strains respectively. The class1 integrons were detected in 2 isolates with the presence of *aadA7* gene cassette in these integrons.

**Conclusion:**

The endemic clonal dissemination and multi-drug resistance of CRPA isolates in our institution is highly alarming. Strict measure will be required to control the further spread of these pathogens in hospital setting.

## Background

*Pseudomonas aeruginosa* is one of the major opportunistic and nosocomial pathogen that causes many severe and often fatal infections, especially in immunocompromised patients or those with underlying diseases [1]. Multidrug resistant *P. aeruginosa* isolates have been detected in hospitals worldwide and associated with increased mortality and costs due to prolonged hospitalization, need of surgery, and prolonged treatment with antibiotics [2]. Increasing resistance in *P. aeruginosa* isolates complicates the selection of adequate empirical therapy in severe infections. Carbapenems are potent broad spectrum β-lactam antibiotics, and one of the few remaining agents that have reliable activity against *P. aeruginosa* [1–3]. However, increased prevalence of resistance to carbapenems among these organisms has been noted [2]. Resistance against carbapenems by *P. aeruginosa* may occur through different mechanisms including: loss of the outer membrane porin OprD protein, reduced levels of drug accumulation due to efflux-pumps over-expression, and increased production of AmpC β-lactamases [1]. Also, *P. aeruginosa* may obtain genes encoding carbapenemase enzymes such as metallo-β-lactamases (MBLs) or *Klebsiella pneumoniae* carbapenemase (KPC) [1, 4]. The MBL enzymes (IMP, VIM, SPM, GIM, SIM, AIM, FIM, and NDM) are able to hydrolyze carbapenems efficiently and thus they are considered as the most clinically significant mechanism of carbapenem resistance in *P. aeruginosa* isolates [4]. VIM-type MBLs are predominant in the Mediterranean region [5, 6]. At Annaba hospital in Algeria and since December 2010, VIM-2 producing *P. aeruginosa* has been isolated, mainly in surgery and intensive care unit [7, 8]. In 2012, an increasing rate of imipenem resistance in *P. aeruginosa* was observed in Annaba hospitals. The effect of carbapenem resistance on the outcome of *P. aeruginosa* infections and risk factors for acquiring carbapenem resistant *P.aeruginosa* (CRPA) in Algeria are unclear. Consequently, the primary objective of the study was to determine the prevalence and molecular characterization of CRPA isolated from our institution. A secondary objective of the study was to identify risk factors associated with carbapenem resistance. It is anticipated that an improved understanding of the prevalence, mechanism, and risk factors of carbapenem resistance in *P. aeruginosa* may guide formulary decisions and the choice of empiric therapy for nosocomial infections in hospitals.

## Methods and materials

### Setting

The study was performed at Annaba university hospital in Annaba city, Algeria. It is one of the major teaching hospitals in the eastern part of Algeria and comprised three affiliated hospitals: Ibn Sina, Dorban and Ibn Rochd. These three facilities included a 279-bed community hospital, a 466-bed hospital dedicated especially to surgical specialities, and the last one with 198-beds, which serve a diverse spectrum of patients.

### Data collection

All the patients infected by *P. aeruginosa* from January, 2012 to December, 2013 were included. The medical records of these patients were retrieved and reviewed. Information was obtained about basic demographic characteristics (age, sex, pre-infection hospital stay, and nosocomial origin) as well as co-morbid diseases (surgical intervention, renal diseases, respiratory diseases, central nervous diseases, and others), presence of previous antibiotic use, use of urinary catheters, intensive care unit admission, previous hospitalization, recent surgery, and length of hospital stay. The diagnosis of nosocomial infection was established according to the Center for Disease Control (CDC) criteria.

Previous hospitalization was defined as hospitalization at Annaba University or another hospital within 30 days prior to the current admission. Recent surgery was defined as any surgical procedure performed in the operating room within 30 days of entry in the study. The origin of the isolate was accepted as nosocomial if the strain was isolated more than one week after hospitalization.

Microbiological specimens were collected when the attending physician suspected infection based on systemic signs (unexplained fever, chills, and hypotension), and/or local signs (purulent tracheal aspirates in mechanically ventilated patients, purulent urinary drainage, or pus or pain at a vascular catheter insertion site). Microbiological specimens were collected as recommended by the CDC. Specimens consisted of blood for bacteremia/septicemia, urine for urinary tract infection, a barncheoalveolar lavage fluid or endotracheal aspirate for ventilator associated pneumonia and purulent discharges, aspirated pus or drain fluid for surgical site infection. *P. aeruginosa* isolated from clinical specimens were identified using conventional methods as well as commercial identification kits, API 20NE (Biomerieux, Marcy l’Etoile, France).

The relationship between CRPA strains and the previous antibiotic therapy was assessed. The antibiotics were grouped as carbapenems, third-generation cephalosprins, quinolones, and others. Previous antibiotic therapy was defined as any systemic antibiotic given at least seven days within 3 months preceding the isolation of the organism. In cases of recurrent episodes of *P. aeruginosa* infections, only the first event was entered into the database.

### Antibiotic susceptibility testing

Antimicrobial drug susceptibility was determined using the disk diffusion method on Mueller-Hinton (MH) agar plates (Bio-Rad, Marnes-la-Coquette, France) according to the recommendations of the Clinical and Laboratory Standards Institute (CLSI, 2012) [9]. Ten antibiotics were tested, including ticarcillin, piperacillin, ticarcillin/clavulanic acid, ceftazidime, imipenem, aztreonam, amikacin, gentamicin, ciprofloxacin, and colistin. *P. aeruginosa* ATCC 27853 was used as a wild-type susceptible control.

Minimum inhibitory concentrations (MICs) of imipenem were determined using an Etest-strip (AB BioMerieux, France), as described by the manufacturers’ instructions.

### Phenotypic detection of the carbapenemase production

The phenotypic detection of the carbapenemase production was performed by the modified Hodge test by using an imipenem disc (10 μg) as was described by CLSI. The detection of metallo-β- lactamase production was also performed by the combined-disc test by using two imipenem discs (10 μg), one containing 10 μL of 0.1 M (292 μg) anhydrous EDTA (Sigma Chemicals, St. Louis, MO), which were placed 25 mm apart on a MH agar plate. An increase in the zone diameter of >4 mm around the imipenem-EDTA disc as compared to that of the imipenem disc alone was considered as positive for metallo-β-lactamase production.

### Phenotypic detection of the AmpC and ESBL production in CRPA isolates

Extended spectrum β-lactamase (ESBL) production was detected by the double-disc synergy test (DDST) using clavulanic acid-ticarcillin (20/10 mg) and ceftazidime (30 mg) and aztreonam (30 mg) on MH agar as described by Hakemi Vala et al. [10]. Phenotypic detection of ESBLs can be obscured by the chromosomal AmpC cephalosporinase in *P. aeruginosa*, hence cloxacillin-containing DDST was performed. Cloxacillin (250 μg/mL; Sigma, St Louis, MO, USA) was added in MH agar to inhibit cephalosporinase activity [11].

AmpC overproduction was confirmed according to the method of Rodrίguez-Martίnez et al. [11]. The isolates were considered as AmpC overproduction when there was at least a twofold dilution difference between the MICs of ceftazidime, imipenem and the MICs of ceftazidime, imipenem plus cloxacillin [11].

### Preparation of DNA template for PCR

Total DNA was extracted by suspending a few colonies of overnight culture of *P.aeruginosa* isolates growing on Luria Bertani agar (Bio-Rad, Marnes-la-Coquette, France) in 500 μL of DNase- and RNase-free water (Invitrogen, Paisley, UK). The suspension was boiled at 100 °C for 10 min in thermal block (Polystat 5, Bioblock Scientific, France), then centrifuged at 19000 x g for 5 min. An aliquot of 1 μL of the supernatant was used as the DNA template for PCR.

### Detection of β-lactamases encoding genes

All CRPA isolates were screened by PCR for the following carbapenem hydrolysing enzymes encoding genes: *bla*_VIM_, *bla*_IMP_, *bla*_GES_, *bla*_KPC_, *bla*_OXA-58,_*bla*_OXA-40_, *bla*_OXA-23_ and *bla*_OXA-51_ and others β-lactamases : *bla*_TEM_, *bla*_SHV_, *bla*_SPE-1_ and *bla*_OXA-1_ as described previously [8, 12, 13]. CRPA resistance to ceftazidime antibiotic was further tested by using primers specific for plasmid mediated AmpC β-lactamase encoding genes as described by Pérez-Pérez and Hanson [14].

### Detection of class 1 integrons

PCR was performed using primers CS-3’ and CS-5’ to amplify different fragment size of class 1 integrons. The primers, PCR conditions and reaction mixtures used were as described previously [12].

### Detection of plasmid-mediated quinolone resistant, and aminoglycoside resistant- genetic determinants

The assessment of plasmid mediated quinolone resistance (PMQR) and aminoglycoside resistant determinants carried by CRPA was conducted as described previously [13, 15]. All CRPA strains were screened by multiplex PCR for *qnr* genes (*qnrA*, *qnrB* and *qnrS*). PCR amplification of *aac(6’)-Ib* encoding aminoglycoside 6′-N-acetyltransferase type *Ib* enzyme and *aac(3)-II* encoding *3*-N-aminoglycoside acetyltransferases genes was performed using primers that amplify all variants.

### Sequencing of resistance genes

All amplified products obtained were sequenced to validate their identities. Both strands of the purified amplicons were sequenced with a Genetic Analyzer 3130x1 sequencer (Applied Biosystems, Foster City, CA, USA), with the same primers used for PCR amplification. The nucleotide and deduced protein sequences were analyzed with the software available over the Internet at the National Center for Biotechnology Information website (www.ncbi.nlm.nih.gov).

### Genotyping of CRPA isolates by Pulsed-field gel electrophoresis

Pulsed-field gel electrophoresis (PFGE) analysis was performed according to the standardized protocol developed by Durmaz et al. [16] using *Spe*I. The Dice similarity coefficient was calculated between pairs of lanes, and the strains were grouped, using the dendrogram construction utility Dendro UPGMA (Biochemistry and Biotechnology Department, Rovirai Virgili University, Tarragona, Spain) (http://genomes.urv.cat/UPGMA/index.php). The isolates were considered to be genetically related if the Dice coefficient of correlation was 80 % or greater.

### Analysis of data

To identify variables associated with CRPA, a risk factor analysis was performed using a case–control study format. Demographics and hospitalization variables of CRPA case patients were compared with patients with carbapenem susceptible *P. aeruginosa* (CSPA). Data were entered into a database using the SPSS 20.0 for Windows (SPSS Inc, Chicago, USA). The *χ*^2^ test and the independent samples *t* test were used for categorical and continuous variables, respectively. A stepwise multivariate logistic regression was conducted to examine the association of risk factors controlling for potential confounders. The logistic model included all variables for which a *p* value of < 0.1 was obtained in the multivariate analysis. A *p* value of <0.05 was considered as significant.

## Results

A total of 80 patients were included in this study and distributed in 40 % female and 60 % male (M:F ratio was 1.5). Fifteen patients presented CRPA infection (18.75 %; 95 % confidence interval: 10.2-27.3) and 65 patients had CSPA infection. The median age was 45 years-old in the CRPA group, and 51 in the CSPA group, with no significant difference, using parametric (for mean) or non-parametric (for median) tests. The duration of hospitalization was different between the groups, although it had a tendency to be prolonged in the CRPA group, with a median of 63.44 ± 48.82 days in the CSPA group and 82.5 ± 45.82 days in the CRPA group. The infected patients were hospitalized in various hospital units with the majority of patients from the endocrinology (55 %), followed by the patients from the otorhinolaryngology (18.75 %) and surgery (11.25 %) wards, while the rest of the patients were from various other hospital wards. According to anatomical location, the infections caused by *P. aeruginosa* were divided into infections of skin, surgical site infections, and soft tissues (70 %), respiratory tract infections (17.50 %), urinary tract infections (11.25 %), and bloodstream infections (1.25 %). All the clinical data are detailed in the Table 1. Even though several variables were compared, the urinary catheter use, previous use of antibiotics, and co-infection by *Staphyloccocus aureus* were statistically significant. In the multivariate analysis none of the risk factor for CRPA was identified. The treatment of *P. aeruginosa* infection was incorrect in 57 of 80 patients (71.20 %) (Table 2). The mortality of patients with *P. aeruginosa* infection was 1.53 % for patients with CSPA and 13.34 % for patients with CRPA, without statistical significance.

**Table 1:** Characteristics of patients infected with carbapenem-resistant *P. aeruginosa* and carbapenem-susceptible *P. aeruginosa*

Variable	CRPA (n = 15)	CSPA (n = 65)	*p*-value
Age-years			
Mean	47.54 ± 12.7	47.45 ± 20.88	0.5
Gender–n (%)			
Female	5(33.34)	27(41.53)	0.57
Male	10(66.66)	38(58.46)	0.60
Sexe-ratio	0.5	0.68	
Duration of hospitalization-days	82.5 ± 45.82	63.44 ± 48.82	0.04
Ward			
Otorhinolaryngology	0	15(23.07)	0.06
Endocrinology	9(60)	36(53.85)	0.74
ICU-surgery	3(20)	6(9.23)	0.36
ICU-medecin	0	4(6.15)	1
Outpatient	3(20)	4(6.15)	0.12
Source			
Diabetic ulcers	9(60)	47(70.76)	0.34
Urine	4(26.66)	5(7.7)	0.08
Blood	1(6.66)	0	0.18
Aspiration tracheal	1(6.66)	13(20)	0.45
Coexisting diseases			
Trauma	3(20)	6(9.23)	0.36
Diabetes mellitus	7(46.66)	35(53.85)	0.5
Otitis	0	7(10.77)	0.03
Respiratory disease	0	4(6.15)	1
Renal disease	0	2(3.07)	1
Other	0	6(9.23)	0.5
Previous antibiotics use	11(73.34)	59(90.76)	0.03
Cephalosporin (third-generation)	10(66.67)	44(67.69)	1
Aminoglycoside	1(6.66)	12(18.46)	0.4
Fluroquinolone	0	2(3.07)	1
Without antibiotic therapy	4(26.66)	7(10.77)	0.2
Invasive devices	3(20)	12(18.46)	1
Urinary catheter	3(20)	2(3.07)	0.04
Intubation	0	5(7.7)	0.5
Intubation + urinary catheter	0	5(7.7)	0.5
Polymicrobial infection	9(60)	33(50.77)	0.5
*K. pneumoniae*	1(6.66)	1(1.53)	0.3
*S. aureus*	5(33.34)	6(9.23)	0.01
*E. coli*	2(13.33)	8(12.30)	1
*S. aureus* + *E. coli*	1(6.66)	4(6.15)	1
*S. aureus* + *P. mirabilis*	0	10(15.38)	0.19
*S. aureus* + *K. pneumoniae*	0	3(4.61)	1
Mortality	2(13. 33)	1(1.53)	0.09

CRPA:carbapenem-resistant *P. aeruginosa*, CSPA: carbapenem-susceptible *P. aeruginosa*

**Table 2:** β-lactams administered to patients

β-lactams		Patient infected by CRPA	Patient infected by CSPA	*p-*value
Cephalosporin (third-generation				
	Cefotaxime (calforan®)	10	42	0.9
	Cefexime	0	1	1
	Ceftriaxone	0	1	1
Carbapenem				
	Imipenem (Teinam ®)	3	0	0.005

CRPA:carbapenem-resistant *P. aeruginosa*, CSPA: carbapenem-susceptible *P. aeruginosa*

The antimicrobial susceptibility testing was done by disc diffusion method to 80 clinical isolates of *P. aeruginosa*. The resistant rates for β-lactam antibiotics including ticarcillin, ceftazidime, ticarcillin/clavulanic acid, aztreonam and piperacillin were 61.25 %, 56.25 %, 56.25 %, 41.25 % and 36.25 %, respectively. The resistant rates for non-β-lactam antibiotics including ciprofloxacin, colistin and gentamicin were 43.75 %, 25 %, and 21.75 %, respectively. The degrees of antibiotic resistance for all antibiotics tested were significantly higher in the CRPA isolates, compared with CSPA isolates (Table 3). Colistin and amikacin were the most effective antibiotics against CRPA isolates with a susceptibility of 93.90 % and 100 % respectively. CSPA isolates were significantly more susceptible to ticarcillin/clavulanic acid (51.56 % vs. 7 %), aztreonam (70.31 % vs. 7 %), gentamicin (46.66 vs. 15.38 %) and ceftazidime (50 % vs. 13 %) compared to CRPA isolates.

**Table 3:** Comparison between CRPA and CSPA on the susceptibility of antibiotics tested

Antibiotics	Proportion of resistance (%), (No. of resistant strains)		*p*-value
	CRPA	CSPA	
Ticarcilline	100 % (15)	53 % (34)	>0.0001
Ticarcilline + clavulanic acid	93 % (14)	48.44 % (31)	0.001
Ceftazidime	87 % (13)	50.00 % (32)	0.008
Aztreonam	93 % (14)	29.69 % (19)	>0.0001
Piperacilline	87 % (13)	25.00 % (16)	>0.0001
Ciprofloxacine	100 % (15)	31.25 % (20)	>0.0001
Colistin	7 % (4)	24.61 (16)	1
Amikacin	0 %	0 %	
Gentamicin	46.66 % (7)	15.38 % (10)	0.007

CRPA:carbapenem-resistant *P. aeruginosa*, CSPA: carbapenem-susceptible *P. aeruginosa*

**Fig. 1:**
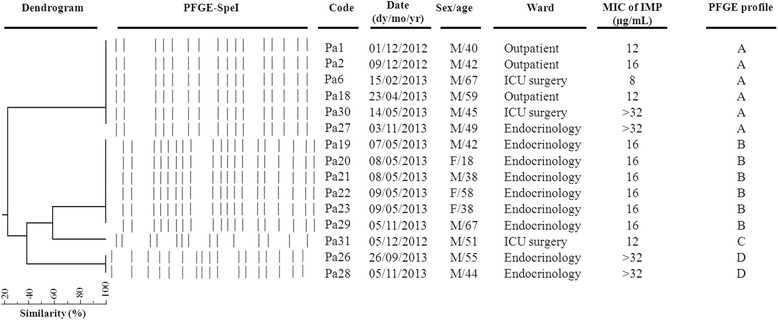
Representative *Spe*I pulsed-field gel electrophoresis (PFGE) profiles of carbapenem–resistant *P. aeruginosa* isolates studied. A dendrogram was generated with Dendro UPGMA ((http://genomes.urv.cat/UPGMA/index.php). The PFGE profile, the sex and age of patients infected, and wards are indicated

The MIC values for the imipenem varied widely among the CRPA isolates. The ranges of the MIC values were 8- > 32 μg/mL (Fig. 1). MBL screening with EDTA and modified Hodge test was negative in all isolates. PCR analysis was performed for all the CRPA isolates. None of the isolates gave positive PCR results for carbapenemase encoding genes; *bla*_VIM_, *bla*_IMP_, *bla*_GES_, *bla*_KPC_, *bla*_OXA-58,_*bla*_OXA-40,_*bla*_OXA-23_ and *bla*_OXA-51_. Synergy tests were performed using clavulanic acid-ticarcillin-, aztreonam-, and ceftazidime-containing disks did not give evidence of any inhibition of aztreonam and ceftazidime resistance for all the CRPA isolates, ruling out the production of ESBL. PCR detecting of carbenicillinase encoding gene *bla*_PSE-1_ was positive in two isolates (P.a29 and P.a30). AmpC over-expression was previously reported to be highly correlated with ceftazidime resistance. We found that AmpC over-expression was present in 62 % of CRPA isolates and none gave positive PCR results for plasmid-mediated AmpC β-lactamase genes. Among seven gentamicin resistant CRPA isolates, five harboured *aac(3)-II* encoding *3*-N-aminoglycoside acetyl-transferases gene (P.a1, P.a2, P.a18, P.a29, P.a30). The plasmid-mediated quinolone resistance genes, was not found in the examined isolates. The class 1 integron (*Int1*) was detected in two isolates (P.a29 and P.a30) with PCR amplicons 1 kb in length. Sequence analysis of these amplicons revealed the presence of the *aadA7* gene cassette.

In the genotyping analysis, the DNA fingerprint patterns of the 15 carbapenem-resistant isolates revealed four distinct clones, as shown in Fig. 1. One of these clones was represented by a single isolate (isolate P.a31, clone C). The other two clones were represented by six (clone A and B), and two (D) isolates, respectively. Clone A and B were isolated from two different hospitals. Clone A came from two different wards outpatients and ICU wards, showing a wide MIC range (8- > 32 μg/mL), and clone B which exclusively came from endocrinology ward, showed a homogeneous MIC value (16 μg/mL).

The combination of PFGE results with hospital overlap data in each ward, demonstrated from patient -to-patient transmission in 8 (53.34 %) patients at endocrinology ward (clone B and D). In contrast, the same isolates of other clones were found in patients hospitalized in the same ward within a few months of each other, indicating that the persistence of the clone at the ward or hospital is still existing.

## Discussion

Carbapenems are among the best choices for the treatment of infections caused by multi-drug*-*resistant *P. aeruginosa* (MDR- *P. aeruginosa*) isolates [1]. In recent years, Algeria has been considered among the countries that reported high rates of antimicrobial resistance [7, 8, 17]. In the present study, there were high levels of resistance to all commercially available antimicrobial agents among *P. aeruginosa* isolated from Annaba Hospital; the rate of 18.75 % CRPA isolates, this rate of carbapenem resistance reflects a threat limiting the treatment options in our hospitals. The rates of CRPA isolates varied by geographic region, specimen source, and selective pressure from antibiotics [18]. In Algeria, Drissi et al. [17] concluded that *P. aeruginosa* isolates exhibited the highest resistance levels to imipenem (35 %) in the period between 2005–2007. Also Sefraoui et al. [8] showed that among *P. aeruginosa* strains 39.32 % were resistant to imipenem during the period 2009–2012. However, the CRPA frequency shown in the studied hospitals during the period 2012–2013 was lower than those reported in Algeria. Among the neighboring countries, such as Libya, Tunisia and Egypt the occurrence of imipenem resistant *P. aeruginosa* was reported often and it ranged between 24.2 % and 39.34 % [19–21].

In our study, CRPA were more resistant to multiple drugs than CSPA isolates, and the most effective antibiotic against CRPA isolates was amikacin and colistin. These findings indicate that amikacin and colistin has increasingly become the last viable therapeutic option for MDR-*Pseudomonas* infections. The high percentage of co-resistance to carbapenem and fluroquinolone is relevant among the studied CRPA, highlighting the percentage of resistance to ciprofloxacin (100 %) that was higher than other previous studies [8, 17, 21]. Carbapenem resistance in *P. aeruginosa* strains may result from multiple mechanisms with or without the production of carbapenemase [1]. Loss or under expression of porin OprD is the most common mechanism of resistance to carbapenems and is frequently associated with efflux pumps and/or AmpC over expression [1, 22]. In our study, none of the CRPA isolates found were positive for carbapenemase-producing genes, and the MIC values for imipenem ranged between 8- > 32 μg/mL. These results suggested the presence of other mechanisms such as over-expression of the efflux pump or loss of outer membrane porin. In addition, the mutational disruption of *oprD* is the major emerging mechanism of carbapenem resistance among *P.aeruginosa* isolates in Algeria [7, 8]. Several previous studies have examined the occurrence of aminoglycoside resistance mechanisms in *P. aeruginosa* isolated from different countries [1, 23, 24]. In our study, the rate of gentamicin resistance found in CRPA (46.6 %) was much higher than the rate that has been reported previously in Annaba hospitals (28.09 %) [8]. The *aac(3)-II* was the only resistance gene observed in this study. This result was in accordance with what has been observed in different studies in other countries, in which the transferable aminoglycoside-modifying enzymes were the most frequent mechanisms in aminoglycoside resistance in *P. aeruginosa* [25, 1, 26]. The class I integron and carbenicillin hydrolysing β-lactamases of *Pseudomonas* specific enzyme (PSE-1) type were found in two CRPA isolates. These class I integrons may play an important role in the development of antimicrobial resistance and emergence of MDR- *P. aeruginosa* [27].

The risk factors for acquiring CRPA may be related to the host condition, infection control practice, and antimicrobial consumptions [28–30]. Regarding invasive procedures, in our study urinary catheter is hypothesized to be a risk factor for CRPA- infection. This was not surprising, given that *P.aeruginosa* tends to make biofilm on the surface of urinary catheters, and they increase the risk of translocation of organisms to the urinary tract, causing infection [18, 30, 31]. These results highlight the need for improved measures to control nosocomial infection and show that the manipulation of invasive devices is one of the main procedures that require intervention measures.

The length of hospital stay before isolation of CRPA was also reported as the risk factor in this study. This result is consistent with many studies evaluating the relationship between acquisition of CRPA infection and the length of stay [28, 30, 32, 33]. One possible explanation for these findings is that patients who are in hospital for longer periods of time have increased exposure to nosocomial pathogens and, subsequently, are at increased risk of being colonized by these organisms [28–30, 32].

A clonal dissemination of CRPA isolates with the presence of two important CRPA clones were observed in this study. In our study, we defined cases of patient-to-patient transmission on the basis of isolates with similar PFGE patterns and an overlap in hospital stay. These cases which were observed in 53.34 % patients at endocrinology ward (clone B and D), suggest that patient-to-patient transmission is an important dissemination mechanism and has contributed to the increased rate of resistance to carbapenems. The patients admitted in endocrinology wards often have compromised immune systems (all patients harboured diabetes mellitus disease) receiving excessive manipulation (such as catheterization, intubation, blood collection, etc.) and a high antimicrobial intake, which could have been predisposing factors for infections and the spread of resistant bacteria [30].

In parallel with other services presented in this study, the spread of the pathogen can be explained by the fact that in hospitals where the most serious infections occur, *Pseudomonas* can be spread too, on the hand of healthcare workers or by an equipment that gets contaminated and is not properly cleaned. These results suggest an exogenous, preventable acquisition of *P. aeruginosa*, which should stimulate research on policies of premises decontamination and their impact on *P. aeruginosa* acquisition.

The differences between services can be explained by differences in patient population and by differences in the implementation of general measures of hygiene to control cross-transmission between patients.

Patients in the current study were already hospitalized in other wards and other hospitals; the movements of patients between different hospital wards must therefore also be considered.

### Limitations

As with any retrospective study, our study is not without limitations, for the nature of retrospective design, the diagnosis and management of sepsis and antibiotic choice were based on individual clinicians’ opinions. Our data were collected from a single site, so institutional differences in prescribing patterns, antibiotic formularies, and patient populations may affect the applicability of our results to other institutions. On the other hand, because active surveillance was not performed over the study period, we cannot ensure that control patients did not harbour CRPA. Our small sample size was another limitation; it may have limited the detection of other risk factors. Finally, the completed molecular characterization of carbapenem resistance mechanisms in CRPA isolated in this study, such as the membrane permeability and efflux mechanisms should be investigated.

## Conclusion

In conclusion, to the best of our knowledge, this is the first report to identify risk factors in Algeria for *P. aeruginosa* resistant to carbapenem. Our study confirmed that the length of hospital stay is the major risk factor for CRPA, as the same presence of invasive devices.

In addition, our result also demonstrate clonal dissemination of CRPA isolates, suggest cross-transmission as an important dissemination mechanism and has contributed to the increased rate of resistance to carbapenems. A clear understanding of risk and the mechanism of carbapenem resistance prevalent in a hospital is vital to devise tailor-made intervention strategies also; effective empiric therapy for nosocomial infections can be rationally formulated.
